# Zoonotic *Cryptosporidium* species and subtypes in lambs and goat kids in Algeria

**DOI:** 10.1186/s13071-018-3172-2

**Published:** 2018-11-06

**Authors:** Djamel Baroudi, Ahcene Hakem, Haileeyesus Adamu, Said Amer, Djamel Khelef, Karim Adjou, Hichem Dahmani, Xiaohua Chen, Dawn Roellig, Yaoyu Feng, Lihua Xiao

**Affiliations:** 1 École Nationale Supérieure Vétérinaire, Rue Issaad Abbes, El Alia, Alger, Algérie; 2 Division of Foodborne, Waterborne and Environmental Diseases, Centers for Disease Control and Prevention, 1600 Clifton Road, Atlanta, GA 30329 USA; 3 Laboratoire exploration et valorisation des écosystèmes steppique, Université Ziane Achor, 17000 Djelfa, Algérie; 4 Department of Biology, Addis Ababa University, Addis Ababa, Ethiopia; 5 Department of Zoology, Faculty of Science, Kafr El Sheikh University, Kafr El Sheikh, 33516 Egypt; 6 UMR-BIPAR, ANSES-Ecole Nationale Vétérinaire d’Alfort, Maisons-Alfort, Paris, France; 7 Université Saad Dahleb Blida, Blida, Algérie; 8 Beijing Tropical Medicine Research Institute, Beijing Friendship Hospital, Beijing, 100050 China; 9 Key Laboratory of Zoonosis of Ministry of Agriculture, College of Veterinary Medicine, South China Agricultural University, Guangzhou, 510642 China

**Keywords:** *Cryptosporidium parvum*, *Cryptosporidium ubiquitum*, *Cryptosporidium xiaoi*, Goat, Sheep, Algeria

## Abstract

**Background:**

Little is known on the occurrence and identity of *Cryptosporidium* species in sheep and goats in Algeria. This study aimed at investigating the occurrence of *Cryptosporidium* species in lambs and goat kids younger than 4 weeks.

**Methods:**

A total of 154 fecal samples (62 from lambs and 92 from kid goats) were collected from 13 sheep flocks in Médea, Algeria and 18 goat flocks across Algiers and Boumerdes. They were screened for *Cryptosporidium* spp. by nested-PCR analysis of a fragment of the small subunit (*SSU*) rRNA gene, followed by restriction fragment length polymorphism and sequence analyses to determine the *Cryptosporidium* species present. *Cryptosporidium parvum* and *C. ubiquitum* were further subtyped by sequence analysis of the 60 kDa glycoprotein gene.

**Results:**

*Cryptosporidium* spp. were detected in 17 fecal samples (11.0%): 9 from lambs (14.5%) and 8 from goat kids (8.7%). The species identified included *C. parvum* in 3 lambs, *C. xiaoi* in 6 lambs and 6 goat kids, and *C. ubiquitum* in 2 goat kids. *Cryptosporidium* infections were detected mostly in animals during the first two weeks of life (7/8 for goat kids and 7/9 for lambs) and in association with diarrhea occurrence (7/17 or 41.2% goat kids and 7/10 or 70.0% lambs with diarrhea were positive for *Cryptosporidium* spp.). Subtyping of *C. parvum* and *C. ubiquitum* isolates identified the zoonotic IIaA13G2R1 and XIIa subtype families, respectively. Minor differences in the *SSU* rRNA gene sequences were observed between *C. xiaoi* from sheep and goats.

**Conclusions:**

Results of this study indicate that three *Cryptosporidium* species occur in lambs and goat kids in Algeria, including zoonotic *C. parvum* and *C. ubiquitum*. They are associated with the occurrence of neonatal diarrhea.

## Background

*Cryptosporidium* spp. are common enteric protozoa of humans and a wide range of animals [1]. They are involved in numerous outbreaks of diarrheal illness in humans and pre-weaned calves [2, 3]. However, studies of *Cryptosporidium* spp. in small ruminants are much smaller in numbers compared to those in cattle, especially from developing countries [4–6]. Data accumulated thus far indicate that cryptosporidiosis in small ruminants can lead to severe diarrhea, anorexia and weight loss in goat kids and lambs [5, 7–9]. Considerably high infection rates have been reported in these animals in some areas [10–14].

Currently, over 30 *Cryptosporidium* species have been recognized based on morphological, biological and molecular characteristics (reviewed in [1]). Among them, *C. parvum*, *C. ubiquitum* and *C. xiaoi* are common species in small ruminants, although a small number of animals were reportedly infected with other *Cryptosporidium* species such as *C. andersoni* and *C. hominis* [4, 5, 14–18]. Geographical variations in the distribution of these *Cryptosporidium* spp. in small ruminants, however, have been described among the small number of studies conducted [19]. The common occurrence of zoonotic *C. parvum* and *C. ubiquitum* in goats and sheep has raised public health concerns over cryptosporidiosis. While *C. parvum* is well known for causing diarrhea in small ruminants, the pathogenicity of *C. ubiquitum* and *C. xiaoi* remains unclear [2].

Algeria is adopting intensive farming of small ruminants to cope with the high demand for meat and milk. A recent estimate from the Ministère De L'Agriculture et du Développement Rural indicates that the country has approximately 2,800,000 sheep and 490,000 goats (http://www.minagri.dz/contacts.html). Although *Cryptosporidium* spp. from cattle, horses, camels and chickens have been recently characterized using molecular biological tools [20–23], little information is available on the identity of *Cryptosporidium* spp. in goats and sheep. In the present study we therefore generated some preliminary data on the occurrence of *Cryptosporidium* species in goat kids and lambs in Algeria.

## Methods

### Collection of samples

**Fig. 1:**
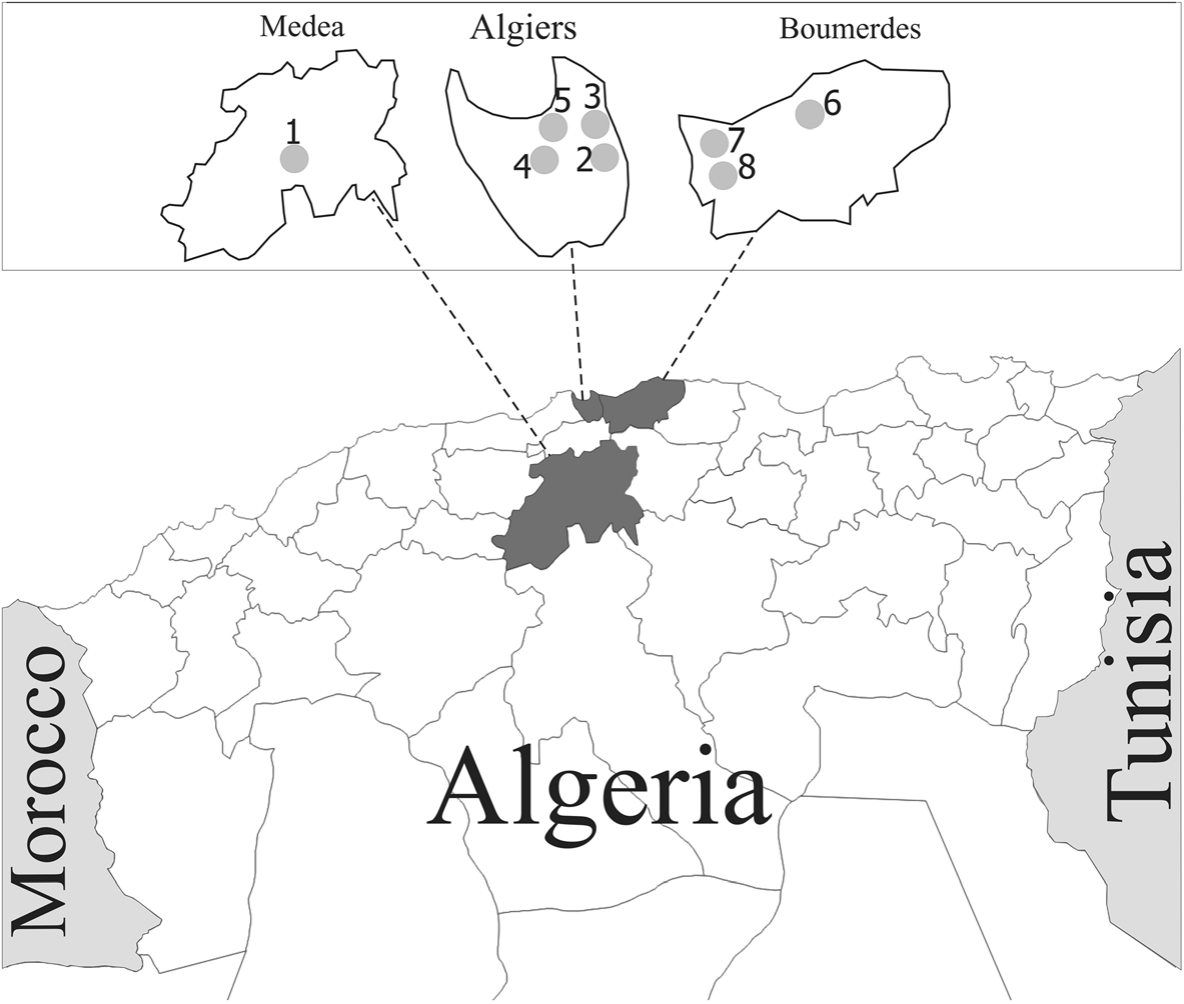
Map of Algeria indicating the locations of sheep and goats in Medea, Algiers and Boumerdes provinces

This study was conducted between January 2012 and January 2014 on 13 sheep flocks in Ksar el Boukhari of Médea Province (No. 1), and 18 goat flocks from seven localities in the provinces of Algiers (Nos 2, 3, 4, 5) and Boumerdes (Nos 6, 7, 8) (Fig. 1). A total of 92 and 62 fecal samples were collected directly from the rectum of goat kids and lambs, respectively. Only animals aged 4 weeks or younger were sampled. Fecal consistency and demographic data on the animals were recorded at the site of sample collection. The samples were transported to the laboratory in ice boxes and preserved in 2.5% potassium dichromate at 4 °C until molecular analysis.

### DNA extraction and PCR analysis

Potassium dichromate was washed off fecal samples with distilled water by centrifugation. Genomic DNA was extracted from 0.2 ml of fecal slurry without further pathogen concentration using the FastDNA SPIN Kit for Soil (BIO 101, MP Biomedicals, Carlsbad, CA, USA). DNA preparations were screened for *Cryptosporidium* spp. by using a small subunit (*SSU*) rRNA-based nested PCR, with DNA of *C. baileyi* as the positive control and reagent-grade water as the negative control. The detection limit of the approach was ~10 oocysts per gram of feces. *Cryptosporidium* species in positive PCR products were determined by restriction fragment length polymorphism (RFLP) analysis using restriction enzymes *Ssp*I and *Mbo*II as described [24] and by DNA sequencing. *Cryptosporidium parvum* and *C. ubiquitum* were subtyped by nested-PCR-sequence analysis of the *gp60* gene as previously described [25, 26].

### DNA sequence analysis

To confirm the identification of *C. ubiquitum* and *C. xiaoi*, the secondary PCR products of the *SSU* rRNA gene from the two *Cryptosporidium* species were sequenced in both directions on an ABI 3130 Genetic Analyzer (Applied Biosystems, Foster City, CA, USA). The *SSU* rRNA gene products of *C. parvum* were not sequenced because it has a well-known *Ssp*I and *Mbo*II RFLP pattern. In addition, all PCR products of the *gp60* gene were sequenced to identify *C. parvum* and *C. ubiquitum* subtypes. The generated sequences were assembled using the ChromasPro v.1.5 software (http://www.technelysium.com.au/ChromasPro.html) and aligned with each other and reference sequences downloaded from GenBank using ClustalX (http://www.clustal.org/)*.* Representative sequences generated in the study were submitted to GenBank under accession numbers LC414387-LC414393 for the *SSU* rRNA gene and LC414394 and JX412917 for the *gp60* gene of *C. parvum* and *C. ubiquitum*, respectively.

### Statistical analysis

*Cryptosporidium* infection rates between diarrheic and non-diarrheic animals were compared statistically using Fisher’s exact test implemented in the Statistical Package for the Social Sciences (SPSS version 22.0). Differences were considered significant at *P* ≤ 0.05.

## Results

### Occurrence of *Cryptosporidium* spp. in goat kids and lambs

*Cryptosporidium* spp. were detected in 8/92 (8.7%) fecal samples from goat kids and in 9/62 (14.5%) fecal samples from lambs, with an overall infection rate of 11.0%. They were present on 3/18 goat farms and 4/9 sheep farms (Table 1). Most *Cryptosporidium*-positive samples were from animals up to 3 weeks of age with diarrhea for both goats and sheep (Table 2).

**Table 1:** *Cryptosporidium* species in goat kids and lambs in Algiers, Boumerdes and Médea provinces, Algeria

Location	No. of farms	Host	No. of samples	No. positive for *Cryptosporidium* (no. of positive farms)	*Cryptosporidium* spp. (no. of samples)	Subtype (no. of samples)
Ksar-el Boukhari (Médea)	13	Sheep	62	9 (4)	*C. xiaoi* (6), *C. parvum* (3)	IIaA13G2R1(3)
Rouiba (Algiers)	3	Goats	16	3 (1)	*C. xiaoi* (3)	–
Bordj-El-Kiffan (Algiers)	2	Goats	11	0	0	–
Heraoua (Algiers)	2	Goats	9	0	0	–
Khemis-elkhechna (Boumerdes)	2	Goats	11	2 (1)	*C. ubiquitum* (2)	XIIa (2)
Hamadi (Boumerdes)	3	Goats	17	0	0	–
Zemouri (Boumerdes)	4	Goats	15	3 (1)	*C. xiaoi* (3)	–
Oued-smar (Algiers)	2	Goats	13	0	0	–
Total	31		154	17 (7)	3 species	2 subtypes

**Table 2:** Occurrence of *Cryptosporidium* spp. in goat kids by age and diarrhea status

Distribution	No. of samples	No. positive for *Cryptosporidium* (%)	*Cryptosporidium* spp. (no. of samples)
By age (days)			
1–7	22	2 (9.1)	*C. xiaoi* (2)
8–14	25	5 (20.0)	*C. xiaoi* (3), *C. ubiquitum* (2)
15–21	27	1 (3.7)	*C. xiaoi* (1)
22–28	18	0	
By age (days) and diarrhea status			
1–7			
Diarrheic	8	2 (25.0)	*C. xiaoi* (2)
Non-diarrheic	14	0 (0)	
8–14			
Diarrheic	9	5 (55.6)	*C. xiaoi* (3), *C. ubiquitum* (2)
Non-diarrheic	16	0 (0)	
15–21			
Diarrheic	4	1 (25.0)	*C. xiaoi* (1)
Non-diarrheic	23	0 (0)	
22–28			
Diarrheic	2	0 (0)	
Non-diarrheic	16	0 (0)	
Total	92	8 (8.7)	*C. xiaoi* (6), *C. ubiquitum* (2)

### *Cryptosporidium* species and subtypes

**Fig. 2:**
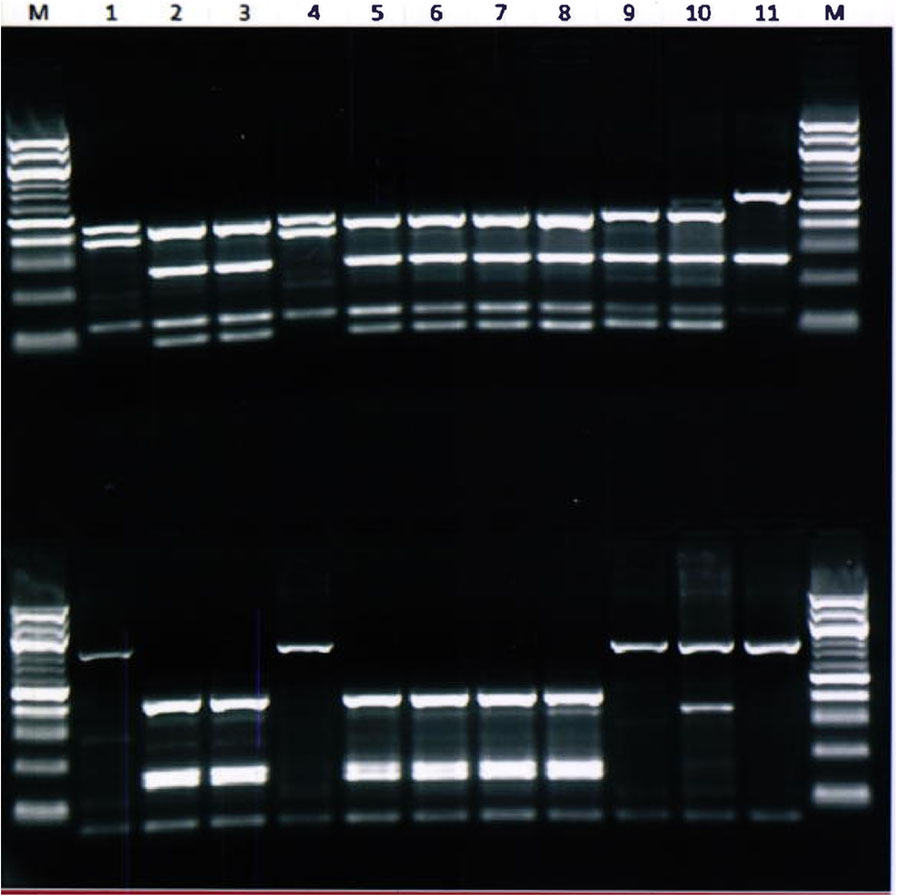
Differentiation of *Cryptosporidium ubiquitum* (Lanes 1, 4), *C. xiaoi* (Lanes 2, 3, 5–8), and *C. parvum* (Lanes 9, 10) from lambs and goat kids by RFLP analysis of the *SSU* rDNA PCR products using restriction enzymes *Ssp*I (upper panel) and *Mbo*II (lower panel). Lane 11: positive control (*C. baileyi*); Lane M: 100 bp molecular markers. The extra *Mbo*II band in Lane 10 is due to the presence of a non-specific band in the PCR product

The RFLP analysis of the *SSU* rDNA PCR products identified two *Cryptosporidium* species in goat kids, including *C. xiaoi* in 6 of 8 *Cryptosporidium*-positive samples and *C. ubiquitum* in 2 of 8 *Cryptosporidium*-positive samples (Fig. 2). *Cryptosporidium xiaoi* was detected in Rouiba (Algiers) and Zemouri (Boumerdes), while *C. ubiquitum* was seen in Khemis-elkhechna (Boumerdes). In lambs, *C. parvum* was present in 3 of 9 *Cryptosporidium*-positive and *C. xiaoi* was identified in the 6 of 9 *Cryptosporidium-*positive samples from Ksar-elBoukhri (Médea). DNA sequencing of *SSU* rDNA PCR products confirmed the detection of *C. ubiquitum* and *C. xiaoi* in these samples.

Data from 12 samples of *C. xiaoi* (six each from sheep and goats) generated two sequence types. The first type was represented by six sequences from sheep and was identical to one *C. xiaoi* sequence (DQ871346) first obtained from a yak in China [24]. The second sequence type was represented by six sequences from goats and was identical to one *C. xiaoi* sequence (EF362478) first obtained from a sheep in the USA [27]. There were two nucleotide differences in the partial *SSU* rRNA gene between the two sequence types (substitution of TT in the first type by CA in the second sequence type at position 420 and 421 of the reference sequence EF362478). The two sequences of the *SSU* rRNA gene fragment of *C. ubiquitum* were identical to each other and to AF442484 initially detected in lemurs in the USA [28].

Sequence analysis of the gp60 gene indicated that the three *C. parvum*-positive samples from lambs had the IIaA13G2R1 subtype, whereas the two *C. ubiquitum*-positive samples from goat kids had the XIIa subtype.

### Occurrence of *Cryptosporidium* spp. by age

In goat kids, *Cryptosporidium* was detected in 2/22 (9.1%), 5/25 (20.0%), 1/27 (3.9%) and 0/18 (0%) of the animals sampled in the first, second, third and fourth week of age, respectively. In contrast, *Cryptosporidium* was detected in 3/15 (20.0%), 4/18 (22.2%), 1/13 (7.7%) and 1/16 (6.3%) lambs sampled in the first, second, third and fourth week of age, respectively. In goat kids, *C. xiaoi* was mostly seen during the first three weeks of age, with two cases (2/22; 9.1%) in the first week, three cases (3/25; 12.0%) in the second week, and one case in the third week (1/27; 3.7%). The two *C. ubiquitum* cases identified in goat kids were seen in the second week of age (2/25; 8.0%) (Table 2). In lambs, one *C. parvum* infection was detected in the first week of age (1/15; 6.7%) and the other two infections in the second week (2/18; 11.1%), while *C. xiaoi* was detected in four animals during the first two weeks (2/15; 13.3% and 2/18; 11.1, respectively) and two animals during the third and fourth weeks (1/13; 7.7% and 1/16; 6.3%, respectively) (Table 2).

### Occurrence of *Cryptosporidium* spp. by diarrhea status

Altogether, 23 of the 92 fecal samples (25.0%) were collected from goat kids with diarrhea, including 8/92 (8.7%) in the first week, 9/92 (9.8%) in the second week, 4/92 (4.3 %) in the third week, and 2/92 (2.2 %) in the fourth week of age (Tables 2, 3). All *Cryptosporidium* infections in goat kids were detected in animals with diarrhea. At the first week of age, *Cryptosporidium* infection was detected in 2/8 (25.0 %) diarrheic kids, with *C. xiaoi* as the only *Cryptosporidium* species involved. At the second week of age, *Cryptosporidium* was observed in 5/9 (55.6 %) diarrheic animals, including *C. xiaoi* (3/5) and *C. ubiquitum* (2/5). At the third week of age, *Cryptosporidium* was present in 1/4 (25.0%) diarrheic ones, with the species being diagnosed as *C. xiaoi*. The occurrence of cryptosporidiosis in diarrheic goat kids (34.8%) was statistically higher compared to that in non-diarrheic (0.0%) ones (*P* = 0*.*0000001).

**Table 3:** Occurrence of *Cryptosporidium* spp. in lambs by age and diarrhea status

Distribution	No. of samples	No. positive for *Cryptosporidium* (%)	*Cryptosporidium* spp. (no. of samples)
By age (days)			
1–7	15	3 (20.0)	*C. xiaoi* (2), *C. parvum* (1)
8–14	18	4 (22.2)	*C. xiaoi* (2), *C. parvum* (2)
15–21	13	1 (7.7)	*C. xiaoi* (1)
22–28	16	1 (6.3)	*C. xiaoi* (1)
By age (days) and diarrhea status			
1–7			
Diarrheic	4	3 (75.0)	*C. xiaoi* (2), *C. parvum* (1)
Non-diarrheic	11	0 (0)	
8–14			
Diarrheic	6	4 (66.7)	*C. xiaoi* (2), *C. parvum* (2)
Non-diarrheic	12	0 (0)	
15–21			
Diarrheic	3	1 (33.3)	*C. xiaoi* (1)
Non-diarrheic	10	0 (0)	
22–28			
Diarrheic	0	0 (0)	
Non-diarrheic	16	1 (6.25)	*C. xiaoi* (1)
Total	62	9 (14.5)	*C. xiaoi* (6), *C. parvum* (3)

Among the 62 fecal samples collected from lambs, 13 (21.0 %) were from diarrheic animals, including 4 in the first week, 6 in the second week, and 3 in the third week of age. *Cryptosporidium* was detected in 3/4 (75.0%), 4/6 (66.7%) and 1/3 (33.3%) of the diarrheic lambs in the first, second and third weeks, respectively (Tables 2, 3). Among them, *C. parvum* was detected in 1/3 and 2/4 of the *Cryptosporidium*-positive samples in the first and second week, respectively. *C. xiaoi* was found in diarrheic animals up to 3 weeks of age and in non-diarrheic ones after that (Table 2). Thus, the overall infection rate in lambs was 14.5%, with the infection rate in diarrheic ones reaching 61.5% (8/13), compared with 2.0% (1/49) in non-diarrheic ones (*P* = 0.0000003).

## Discussion

In the present study, the occurrence and genotype and subtype identity of *Cryptosporidium* spp. in goat kids and lambs in Algeria were examined. The overall infection rate of *Cryptosporidium* spp. was 11.0% (8.7% in goat kids and 14.5 % in lambs). Previous studies reported *Cryptosporidium* infection rates of 5.1–82.0% in sheep and 7.1–93.0% in goats in industrialized nations [4, 10, 14, 16, 29–35]. Few comparable data are available from developing countries, but infection rates of 2.5–67.5 and 2.9–72.5% have been reported in sheep and goats, respectively, in Zambia, Egypt, China, Bangladesh, Iran, Argentina and México [11–13, 17, 36–41]. Variations in infection rates among studies could be attributed to the differences in animal age, diagnostic methods, sample sizes, animal management and climates.

In this study, three *Cryptosporidium* species were identified in small ruminants, including *C. parvum*, *C. xiaoi* and *C. ubiquitum*. In both goats and sheep *C. xiaoi* appeared to be the dominant species (6/8 in goats and 6/9 in sheep), with *C. ubiquitum* being detected only in two of the eight *Cryptosporidium*-positive goats and *C. parvum* in three of the nine *Cryptosporidium*-positive sheep. In concordance with this, *C. xiaoi* was detected as a dominant species in small ruminants in other African countries including Egypt [37] and Tanzania [42] as well as Asian countries such as Bangladesh [40] and China [11, 17, 41, 43]. Similarly, *C. xiaoi* was the major *Cryptosporidium* species in small ruminants in some developed countries such as France [34], Greece [18], Norway [44], Poland [14] and Australia [35, 45]. In the presence study, two types of *SSU* rDNA sequences were obtained from *C. xiaoi*, with sheep and goats having different types. Both sequence types, however, have been observed in both sheep and goats in previous studies based on BLAST analysis of GenBank sequences. As sheep and goat samples from the present study were collected from different areas, it is unclear whether the two types represent different types of *C. xiaoi* circulating in different areas.

*Cryptosporidium parvum* was seen in three sheep among the small number of animals examined. These findings are in agreement with previous common findings of the pathogen in sheep in European countries and Australia [4, 10, 18, 31, 36, 46]. Among developing countries, a small number of *C. parvum* infections have been reported in goats and sheep from Asia, including China [17, 47, 48], India [49], Jordan [50] and Turkey [51]. Our results, however, are in contrast to those from studies conducted in most African countries including Egypt [37], Tunisia [52] and Ethiopia [53], where *C. parvum* has thus far not been reported in small ruminants. *Cryptosporidium parvum* was also absent in sheep and goats in other studies in China [38, 41].

The *C. parvum* identified in the study belonged to the IIaA13G2R1 subtype*.* Although IIaA13G2R1 subtype is not a common *C. parvum* subtype and has not reported previously in sheep, it was detected in some calves in Belgium and Algeria [54, 55], ponies in the USA [56], calves and goat kids in Turkey [51], and humans in Malaysia [57], indicating that it is likely a zoonotic pathogen in a broad range of areas. Similarly, the *C. ubiquitum* in goats in the present study was subtyped as XIIa, a well-known subtype family in goats elsewhere, including Greece [18], China [17, 38] and Australia [45]. It is also commonly reported in sheep in many countries [26, 35, 41]. It is responsible for zoonotic *C. ubiquitum* infection in humans in industrialized countries, especially the UK [26].

In this study, *C. parvum*, *C. xiaoi* and *C. ubiquitum* infections occurred mostly in animals younger than three weeks. This agrees with observations in previous studies [10, 17, 18, 34, 37–40, 47]. Currently, controversy exists on the clinical significance of *C. ubiquitum* and *C. xiaoi* [2]. In the present study, most *C. xiaoi* cases (11/12), the two *C. ubiquitum* and the three *C. parvum* cases all had diarrhea. *Cryptosporidium* infections in lambs and goat kids have been associated with the occurrence of diarrhea in some studies [10, 16, 32, 34, 39, 40]. Case-control studies are needed to confirm the role of *C. ubiquitum* and *C. xiaoi* in the induction of diarrhea in infected animals.

## Conclusions

Results of this study showed a relatively common occurrence of *C. xiaoi* in lambs and goat kids in association with the occurrence of diarrhea. The additional presence of zoonotic *C. parvum* and *C. ubiquitum* indicates that cryptosporidiosis in small ruminants may have further public health implications. More extensive molecular epidemiological studies are needed to substantiate these observations and to improve our understanding of the epidemiology and public health significance of cryptosporidiosis in small ruminants in Algeria.
